# A Functional Food Mixture “Protector” Reinforces the Protective Immune Parameters against Viral Flu Infection in Mice

**DOI:** 10.3390/nu10060743

**Published:** 2018-06-08

**Authors:** Kenza A. Mansoor, Fadi Qadan, Mathias Schmidt, Nidal A. Qinna, Mujtaba Badr, Khalid Z. Matalka

**Affiliations:** 1 Faculty of Pharmacy & Medical Sciences, University of Petra, Amman, Jordan; kmansoor@uop.edu.jo; 2 Herbresearch Germany, Wartbergweg 15, D-86874 Mattsies, Germany; schmidt@herbresearch.de; 3 University of Petra Pharmaceutical Center (UPPC), Amman, Jordan; nqinna@uop.edu.jo (N.A.Q.); mujtaba.badr@usask.ca (M.B.); 4 OncoTherapeutica, Inc., Cambridge, MA 02142, USA; kzmatalka@gmail.com

**Keywords:** pomegranate, red grape, dates, olive fruit, figs, ginger, IFN-γ, IL-12, IL-6, IL-22, flu infection, flu vaccine, hemagglutination inhibition antibodies

## Abstract

**Background**: Viral influenza infection causes serious health issues especially when an outbreak occurs. Although influenza virus vaccines are available and each year manufactures modify the vaccine depending on the expected mutated strain, it is still far from satisfactory, mainly in young children and older adults. Therefore, a product that can support and shape the immune system to protect against viral flu infections is highly essential. **Methods**: A functional food water-soluble mixture of pomegranate, red grape, dates, olive fruit, figs, and ginger extracts, termed herein “Protector”, was prepared and tested in stimulating/modulating the production of specific cytokines, and hemagglutinin inhibition (HAI) antibodies following viral flu vaccination in mice. **Results**: A single intraperitoneal or multiple oral administration for 1–7 days of “Protector” significantly increased the production of interferon (IFN)-γ and interleukin (IL)-12 in blood, spleen, and lungs of mice. When “Protector” was orally administered for one week following a single vaccine injection (primary immunization) or for two weeks (one week apart) following double vaccine injections (secondary immunization), mice significantly produced higher titers of HAI antibodies. This increase in HAI antibodies was associated with Pillow-inducing significant and different changes in vaccine-induced IFN-γ, IL-12, IL-6 and IL-22 following primary and secondary immunizations. **Conclusions**: “Protector” administration reinforces the protective immune parameters against viral flu infection. Therefore, after performing preclinical toxicology studies and ensuring its safety, “Protector” should be considered a potential product to be tested in clinical trials to conclude its efficacy in reducing the devastating effects of flu infection in humans and its outbreaks.

## 1. Introduction

The immune system components including cells, antibodies, cytokines and many other chemical mediators act in orchestric and systemic ways to overcome intruders. Therefore, keeping the immune system vigilant and robust is the factor to eliminate diseases as well as fight infections. Following an inflammatory condition or a viral infection, immune and other tissues produce cytokines to activate and regulate the immune response. During viral infection, cytokines such as type I interferon (IFN), interleukin (IL)-1β, IL-6, and tumor necrosis factor (TNF)-α, are induced following the innate immune response, initiate inflammation via stimulating the production of more cytokines, limit virus replication, and provide signals to activate adaptive immunity [1,2]. Activating the adaptive immunity by producing specific anti-virus antibodies, CD4+ and CD8+ T cells is the ultimate control of virus protection, replication, and clearance [2,3]. For instance, IL-12, a macrophage-derived cytokine, induces T helper (Th)-1 cell activation mainly by producing cytokines that broadly activate immune cells. Of these cytokines, is IFN-γ production. IFN-γ, derived from Th-1 cells, as well as natural killer (NK) cells and dendritic cells (DCs), activate cell-mediated immunity including cytotoxic T cells, promote further DCs, and helps B cells to promote antibody isotype switching [3,4,5]. Furthermore, IFN-γ is a regulator of immune responses, including antiviral defenses [6]. However, the role of IFN-γ as an anti-flu viral cytokine is highlighted by (a) pretreatment with IFN-γ inhibited virus replication, (b) sequential administration of IFN-γ at early stage of the infection protected infected mice from death, and (c) IFN-γ protective effect is distinctive in studies with protocols involving vaccination/transfection followed by viral challenge [7,8,9,10,11,12,13,14]. Therefore, shaping the immune system towards IFN-γ-mediated mechanisms would facilitate clearing the virus and reducing inflammation in the lungs following influenza pathogenesis [9,10,11,12,13,14].

Although IL-6 is an important inflammatory cytokine in the initial phase of influenza viral eradication [1], it has been found to be highly elevated in plasma and hyperactivated from peripheral blood mononuclear cells of patients with complicated viral influenza infection. In addition, IL-6 levels were correlated to intensive care admission [15]. On the other hand, IL-22-derived from NK cells has been shown to have a crucial role in the regeneration of tracheal and bronchial epithelial cells following influenza viral infection in mice [16]. Therefore, regulating/modulating the levels of IL-6 and IL-22 during influenza viral infection may help in reducing the inflammatory-induced pathogenesis and facilitate the construction of the lung epithelial layers.

One of the epidemic viral infections that occur each year; killing hundreds of thousands and hospitalizing millions of people is the flu virus [17]. Influenza virus infections cause a high degree of morbidity and mortality which are related to necrotizing bronchiolitis, diffuse alveolar damage, alveolar hemorrhage, airway obliteration by severe epithelial cell destruction in the lungs and may lead to a cytokine storm that ends up with organs dysfunction. The anti-influenza available drugs are mainly targeting the enzyme neuraminidase that is expressed on the virus surface help to prevent infection as well as exert anti-viral activities [18]. However, drug-resistant strains emerge. Furthermore, although influenza virus vaccines are available and each year manufactures modify the vaccine depending on the expected mutated strain, it is still far from satisfactory mainly in young children and older adults. For instance, the outbreak of flu infection in the winter of 2017/2018 that has occurred in the United States showed that effectiveness of the flu vaccines dropped to 25% whereas it is usually between 40–60% [19]. Therefore, it is highly essential to have a product that stimulates the immune system to protect from viral flu infections as well as reduces or eliminates the devastating counter-response of flu virus that leads to a dysregulated immune response.

One of the essential ways to keep the body’s immune system alert is by eating the appropriate type of food that contains the necessary nutrients and eliminating/reducing the “food” that culminates the immune system. To assist in this, we have developed a water-soluble mixture of edible extracts composed of pomegranate, red grapes, dates, olive fruit, figs, and ginger, termed herein “Protector”. Each extract of this mixture has been shown to modulate the immune system in favor of either stimulating an event or cascade of events in the immune system [20,21,22,23,24,25,26,27,28,29] or reducing inflammatory processes [30,31,32,33,34,35,36,37] that could result during flu infection. The latter anti-inflammatory effect, however, depends on the type of extract (or part of the extract), isolated fraction, and the model used [38,39,40]. Therefore, in order to evaluate the potential of “Protector” in stimulating/modulating the immune system and supporting the protective immune parameters against viral flu infection, we performed several experiments in mice showing its potential to enhance/modulate the production of (a) IFN-γ and IL-12 without and with immunization of the flu vaccine; (b) protective hemagglutinin inhibition (HAI) antibodies following flu vaccination; and (c) the pro-constructive IL-22, and inflammatory-induced IL-6, following flu vaccination.

## 2. Materials and Methods

### 2.1. Fruit Extracts and “Protector” Mixture

All “Protector” fruit extracts were purchased from Naturex, France. Each extract is standardized and quantified based on a specific group of compounds in each extract with complete certificate of analysis. “Protector” consists of pomegranate extract whole dried fruit including peel (400 mg) with drug extract ratio (DER) 5–6:1, dried red grape extract including peel and seeds (200 mg) with DER 10:1, dried dates fruit extract (100 mg) with DER is 2:1, olive fruit dried extract (100 mg) with DER 3–4:1, figs dried extract (150 mg) with DER 2:1, and ginger dried extract (50 mg) with DER 50:1. So in 1 g of “Protector”, the mixture is 4:2:1:1:1.5:0.5.

### 2.2. Animals

The protocols applied were according to the World Health Organization (WHO) for “Evaluating Efficacy and Toxicity of Herbal medicines” and the Federation of Laboratory Animal Sciences Association (FELASA), for Biological and Toxicological studies and were approved by the Ethics Committee at University of Petra. The present animal studies were performed on female Balb/c mice weighing 25 ± 3 g. Animals were housed in a pathogen-free environment at 22 °C with 12 h light/dark cycle at the University of Petra animal care unit. Before each experiment, animals were divided, placed in appropriate cages and were acclimatized for five days to laboratory conditions.

### 2.3. Systemic Administration of “Protector” and Cytokines Levels

For this experiment, mice were divided into five groups (*n* = 4). One negative, one positive control and three treated groups. Animals received a single intraperitoneal (i.p.) injection of 1 mL sterile PBS containing 0.2 µm-filtered amounts (0, 1, 10, 100 µg) of “Protector” or *Eriobotrya japonica* water extract (EJ), as a positive control and a stimulator of IL-12 and IFN-γ [38,39,40]. Twenty-four hours later, mice were sacrificed by cervical dislocation, and each mouse blood sample was directly collected from the cardiac chamber, placed into a pre-chilled tube, weighed and incubated with 2 mL of ice-cold endotoxin-free PBS containing 0.1% Igepal CA-630 under ice for 10 min [41]. After blood collection, spleen and lung tissues were removed from mice, weighed, and processed as the blood. Then tissues were homogenized with a tissue disrupter (Janke and Kundle GmbH, Staufen, Baden-Württemberg, Germany), centrifuged (6000 rpm for 6 min), and the supernatant was transferred to labeled microcentrifuge tubes and stored −30 °C till cytokine assays.

### 2.4. Time Profile of Cytokines Following Systemic Administration of “Protector”

The time profile of cytokines production following systemic administration of “Protector” was evaluated in mice. Thirty-six mice were divided into three groups (16 mice for “Protector” group; 16 mice for PBS group, and 4 mice for zero-time point). Each treated group (groups 2 and 3) received a single i.p. injection of 1 mL sterile PBS (group 2) or a sterile-filtered PBS containing 10 µg of “Protector” (group 3). Following injection, 4 mice/time point (0, 6, 12, 24, and 48 h) were sacrificed and tissues were collected, weighed, and processed as described above.

### 2.5. Oral Dosing of “Protector” and Cytokines Production

Twenty-eight mice were divided into seven groups (12 mice for “Protector” group, 12 mice for PBS group, and 4 mice for zero point). Mice received orally, via a stainless steel oral gavage needle (Harvard Apparatus, Kent, UK), a constant volume of sterile distilled water containing “Protector” (375 µg) or nothing (control groups). Protector was administered three times a day for 1, 3, or 7 days. Group 7 served as a zero-time point. The selection of the dose 375 µg/dose × 3 (3 h apart) (1125 µg/mouse/day), considering mouse average weight is 25 g (i.e., ~45 mg/kg/day), is based on a human dose of ~3000 mg/day (1000 mg/dose). Forty-eight hours post of last administration, mice were sacrificed, and tissues were collected, weighed, and processed as described above.

### 2.6. Influenza Virus Vaccine

In the following experiment, Vaxigrip human vaccine was used. Vaxigrip is a sterile suspension of influenza virus for intramuscular or deep subcutaneous injection. It is a purified, inactivated, split-virion vaccine. It contains the following strains of influenza virus: (1) A/California/7/2009 NYMC X-179A (A/California/7/2009 [H1N1]pdm09-like); (2) A/Victoria/361/2011 IVR-165 (A/Victoria/361/2011 [H3N2]–like); and (3) B/Hubei-Wujiagang/158/2009 NYMC BX-39 (B/Wisconsin/1/2010-like). Each 0.5 mL pre-filled syringe contains 15 µg haemagglutinin of each of the three strains (45 µg in total) in a buffered saline solution. A buffered saline solution consists of the following excipients–sodium chloride, potassium chloride, sodium phosphate–dibasic dihydrate, potassium phosphate–monobasic and water for injection. 

The vaccine is prepared from virus grown in the allantoic cavity of embryonated eggs, concentrated, purified by zonal centrifugation in a sucrose gradient, split by octoxinol 9 (Triton X-100), inactivated by formaldehyde and then diluted in phosphate buffered saline solution to the required concentration. No adjuvant or preservative is added. The vaccine may contain traces of formaldehyde (≤30 µg), octoxinol 9 (≤200 µg) and neomycin (<20 pg). Vaxigrip does not contain more than 0.05 µg ovalbumin per dose.

For preparation of the vaccine, 0.4 mL of sterile PBS was added to each Vaxigrip (45 µg/0.5 mL) vial. This yielded (50 µg/mL) and then a volume of 100 µL/mouse (5 µg of trivalent split-virion vaccine; ~1.7 µg of HA for each strain) was injected subcutaneously (s.c.) in the right thigh of each mouse [42,43].

### 2.7. Immunization Protocol

Primary and secondary immunization protocols were performed using six groups of mice (5 mice/group). The primary immunization protocol was as follows: on day 1, groups 2 and 3 were immunized s.c. with a single injection (5 µg/mouse) whereas group 1 was injected with sterile PBS [43]. Also starting on day 1, mice were orally administered three times a day with 400 µL of sterile distilled water containing 375 µg of “Protector” (group 3) or just water (groups 1 and 2) for 7 days. Mice were kept under observation for another week (i.e., till day 14 post immunization) and then were sacrificed and blood, spleen, and lungs were collected, weighed, and processed as described above. The supernatant was collected into several aliquots and kept frozen until analysis.

As for the secondary immunization protocol, mice (groups 5 and 6) were immunized s.c. with two injections (5 µg each injection/mouse), whereas group 4 with sterile PBS, on days 1 and 14. Also starting on day 1, mice were orally administered three times a day with 400 µL of sterile distilled water containing 375 µg of “Protector” (group 6) or just water (groups 4 and 5) until day 7 and then from day 14 to day 21. Mice were kept under observation for another week (i.e., till day 28 post immunization) and then were sacrificed and tissues were collected as described above.

### 2.8. Hemagglutination Inhibition (HAI) Antibodies Titer Determination

The influenza virus binds to red blood cells (RBCs) and forms agglutination. Thus, having antibodies against the influenza viruses prevents the virus from binding to RBCs and thus no agglutination occurs [42]. Firstly, titration of chicken RBCs with titration of the virus vaccine was performed to establish the percentage of RBCs and amount of virus vaccine to be used. Secondly, serial dilution of mice blood lysates (collected as above) was performed followed by the addition of the diluted virus and incubated for 30 min. After incubation, diluted RBCs solution was added for 1 h. Hemagglutination and precipitation were recorded and visualized under an inverted microscope to determine the titer of each sample.

### 2.9. Cytokine Determinations

Measurements of mouse tissue-extracted cytokines were accomplished by sandwich ELISA developed following the manufacturer’s recommendations (Duoset R & D Systems, Minneapolis, MN, USA). Absorbance values of each well of 96-well plate (Nalge Nunc International, Rochester, NY, USA) were read at 450 nm by GloMax-Multi Detection system (Promega, Madison, WI, USA), transformed to cytokines concentrations (pg/mL) and then normalized to (pg/g of protein/sample).

### 2.10. Statistical Analysis

All data in the figures are presented as the mean ± standard error and assessed by using one-way ANOVA analysis followed by a Tukey’s test for multiple comparisons (SPSS version 17). *p* value of <0.05 was considered significant.

## 3. Results

### 3.1. Systemic Administration of “Protector” Enhanced IFN-γ Production Levels in Blood and Spleen

**Figure 1:**
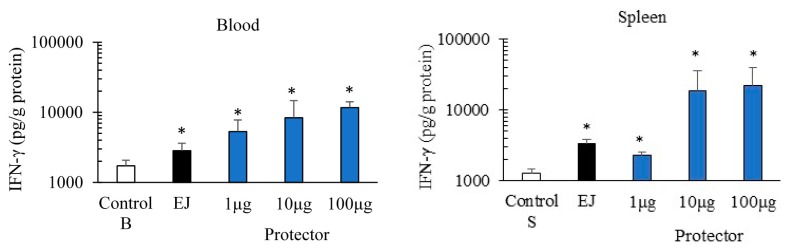
Intraperitoneal administration of “Protector” significantly increased IFN-γ levels in peripheral blood and spleen in dose dependent manner. EJ (10 µg) was used as a positive control. Mice were injected i.p. with “Protector” or EJ and 24 h later, tissues were obtained, processed for cytokines extraction, and then kept frozen until analysis (* indicates *p* < 0.05 in comparison to control mice).

After 24 h of intraperitoneal administration, “Protector” significantly increased the production of IFN-γ in peripheral blood and spleen (Figure 1) in a dose-dependent manner. In comparison to a known inducer of both cytokines (EJ) [38,39,40], 10 µg of “Protector” produced a similar to higher levels of IFN-γ in blood and spleen. A similar pattern of enhancement was observed with IL-12 (data not shown).

**Figure 2:**
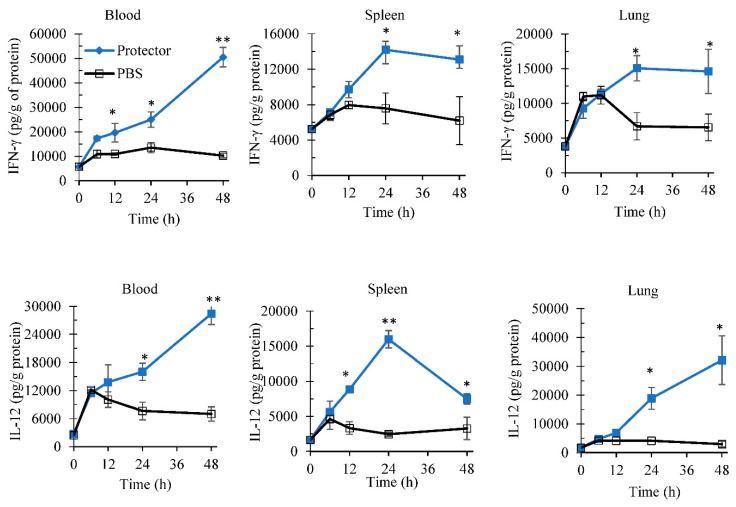
Intraperitoneal administration of “Protector” (10 µg) demonstrated a kinetic production profile of IFN-γ and IL-12 in blood, spleen and lungs. Mice were injected i.p. with “Protector” or sterile PBS, and at different time tissues were obtained, processed for cytokine extraction, and then kept frozen until analysis (* and ** indicate *p* < 0.05 and *p* < 0.01 to their time-point control (PBS), respectively).

To evaluate the time profile of this increase in IFN-γ and IL-12 production, “Protector” at 10 µg dose was administered i.p. in mice. “Protector” significantly increased the production of IL-12 and IFN-γ in blood, spleen, and lungs over time (Figure 2). In comparison to PBS administration (control), “Protector” increased the production of IFN-γ in blood, spleen and lungs after 24 to 48-h post administration. Also, at 12-h post “Protector” administration, blood IFN-γ levels were significantly higher than in PBS group. Similarly, “Protector” administration significantly increased the production of IL-12 in the peripheral blood, spleen, and lungs of mice. This increase was significant at 24- and 48-h post administration for blood and lungs whereas for spleen the increase in IL-12 was significant at 12, 24 and 48-h post administration.

### 3.2. Oral Administration of “Protector” Enhanced IFN-γ and IL-12 Production in Tissues

**Figure 3:**
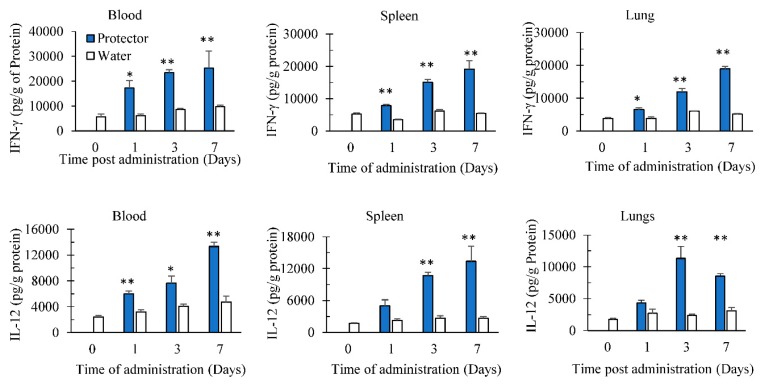
Oral administration of “Protector” for 1, 3 or 7 days significantly increased the production of IFN-γ and IL-12 in blood, spleen and lungs. Mice were orally administered a constant volume of sterile distilled water containing “Protector” (375 µg × 3/day) or nothing (control groups) for either 1, 3, or 7 days. Forty-eight hours later, tissues were obtained, processed for cytokine extraction, and then kept frozen until analysis. (* and ** indicate *p* < 0.05 and *p* < 0.01 to their time-point control (water), respectively).

Before performing the following studies, safety oral dose experiments on “Protector” were completed and found that mice did not show any sign of toxicity for 14 days at the maximum dose tested of 2000 mg/kg (data not shown). Since “Protector” was found to induce cytokines following 24–48 h of intraperitoneal administration and we still need to find if “Protector” gets absorbed in vivo and induces a similar response to intraperitoneal administration, we evaluated “Protector” following multiple oral dosing in inducing cytokines. The target organs tested were peripheral blood, spleen as a secondary lymphoid organ and lungs, as a target of respiratory infections. Oral administration of “Protector” (375 µg × 3/day) significantly increased the production of IL-12 and IFN-γ in blood, spleen, and lungs in comparison to 0-time negative control (group 7) (Figure 3). In comparison to water administration (groups 4, 5 and 6), “Protector” increased the production of Il-12 and IFN-γ in blood, spleen, and lungs after multiple oral dosing for 1, 3 and 7 days. Furthermore, following prolonged multiple dosing i.e., for 7 days, “Protector” significantly produced a higher response than after 1 or 3 days. The sequence of cytokines production response is 7-day dosing >3-day dosing >1-day dosing indicating that not only “Protector” is absorbed and induce immune cells stimulation, but also it has an additive influence on immune cells activation. The latter might indicate that the half-life elimination and or clearance of the active ingredient(s) of “Protector” is/are not short.

### 3.3. Oral Administration of “Protector” Enhanced HAI Antibodies against Influenza Virus

**Figure 4:**
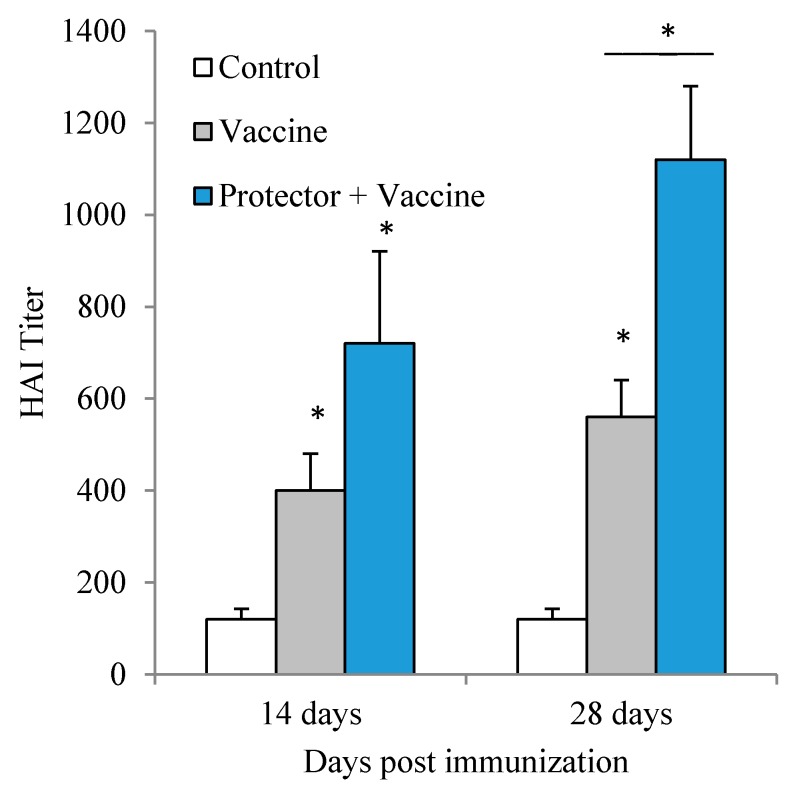
Oral administration of “Protector” increased HAI titer following Vaxigrip primary and secondary immunizations period. Mice received either single s.c. injection of Vaxigrip (5 µg/mouse) at day 1, or two at days 1 and 14. Control mice injected with sterile PBS. Protector was orally administered for 1 week (375 µg × 3/day) for the single immunization group or for 2 weeks (one week apart) for the secondary immunization group (* *p* < 0.05 in comparison to the control and the bar represents the difference between the adjacent groups).

Since “Protector” was found to enhance cytokines production in tissues following systemic as well as multiple oral administration, we further evaluated if “Protector” can enhance HAI antibodies production following immunization with an influenza vaccine. These antibodies are measured as best protective degree against influenza infection in humans [42,43]. Vaxigrip (5 µg/mouse) vaccine significantly induced HAI antibodies against the virus mixture following primary and secondary immunization. However, following secondary immunization, the titer was not significantly different than the primary immunization (Figure 4). When “Protector” was orally administered following primary and secondary immunization periods, HAI antibodies were significantly higher than the Vaxigrip following the secondary immunization period (Figure 4).

**Figure 5:**
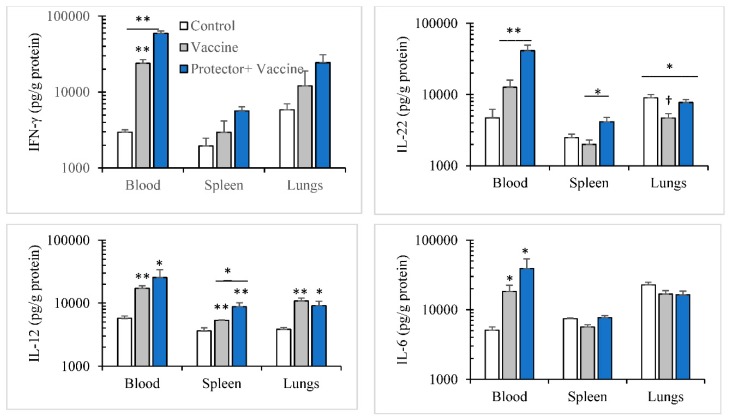
A single/primary immunization with Vaxigrip (5 µg) enhanced the production of IFN-γ, IL-12 and IL-6 in blood as well as IL-12 in spleen and lungs in comparison to PBS injections (Control). When “Protector” (375 µg × 3/day) was orally administered for 1 week, it enhanced Vaxgrip effect on the production of IFN-γ in blood, spleen and lungs; IL-22 in blood and spleen; and IL-12 in spleen. On the other hand, Vazxigrip reduced (†) IL-22 production level in the lungs in comparison to control mice and “Protector” administration restored it. (* *p* < 0.05, ** *p* < 0.01 in comparison to the control group and the bar represents the significance between the adjacent treatments.).

**Figure 6:**
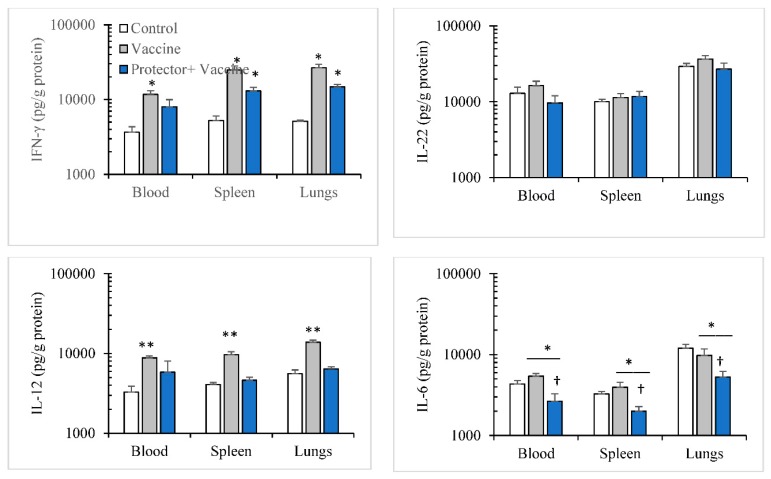
A secondary immunization with Vaxigrip (5 µg/injection at day 1 and 14) enhanced the production of IFN-γ in blood as well as IL-12 in blood, spleen and lungs. When “Protector” (375 µg × 3/day) was orally administered for 2 weeks (one week apart), it modulated Vaxgrip effect on the production of IFN-γ in blood; IL-12 in blood, spleen and lungs; and reduced (†) IL-6 in blood, spleen and lungs. * *p* < 0.05, ** *p* < 0.01 in comparison to the control group and the bar represents the significance between the adjacent treatments.

We also investigated if this increase in anti-influenza hemagglutination antibody is associated with cytokines modulation. The cytokine modulation pattern in blood, spleen, and lungs following primary (14-day period) and secondary (28-day period) immunizations with the Vaxigrip vaccine were similar. The vaccine: (a) significantly increased IFN-γ, IL-12, IL-6 levels in blood; (b) significantly increased IFN-γ and IL-12 in spleen and lungs; but (c) did not modulate IL-6 in spleen or lungs (Figure 5 and Figure 6). However, with “Protector” administration for one week or two weeks (one week apart) during primary and secondary immunizations periods, respectively, the pattern of Protector-induced cytokine modulation were different. “Protector” (a) increased the production of IFN-γ in blood, spleen, and lungs, IL-22 levels in blood and spleen, and IL-12 in spleen more than the vaccine following primary immunization; (b) reversed vaccine-suppressed IL-22 levels in the lungs following primary immunization. Whereas “Protector” administration in the two-time vaccination protocol (a) reversed the vaccine-induced increase in IL-12 in blood, spleen, and lungs; and (b) decreased IL-6 levels in blood, spleen, and lungs more than the control and Vaxigrip groups.

## 4. Discussion

The present study has shown that “Protector” administration in mice enhanced the production of IL-12 and IFN-γ in peripheral blood, secondary lymphoid organ and lungs following intraperitoneal as well as oral dosing. The latter implies that the constituents of “Protector” gets absorbed following oral administration and stimulates immune cells. Such immune cells, for instance, dendritic cells, macrophages, and NK cells are known to produce IL-12 and IFN-γ, respectively [1,2,3,4,5]. Also, NK cells have been found to produce IL-22 [16]. Thus, we can suggest that “Protector” constituents mainly stimulate innate immune cells such as dendritic, macrophages, and NK cells [21,27,29]. It has been known that polysaccharides bind to a variety of toll-like receptors (TLR) on macrophage cell surface receptors, mainly TLR-4, and induce activation [38,39]. However, it has been shown that one of the “Protector” constituents, figs, contains a polysaccharide that enhanced the production of IL-12 and IFN-γ from dendritic cells through a pattern recognition receptors (PRP), dectin-1, not related to TLR, [27]. Furthermore, gingerol, a constituent of ginger, has been found to increase IFN-γ production from activated T cells [29]. Thus, we can suggest the “Protector” constituents indirectly enhance T and B cell functions. In the present study, “Protector” administration enhanced the production of HAI following primary and secondary vaccination with Vaxigrip. Such enhancement could be associated with an increase in cytokines response from DC, macrophages that stimulated T cells and B cells function [21,25].

The kinetic patterns of the IFN-γ and IL-12 productions in blood, spleen, and lungs following a single i.p. injection revealed that these tested cytokines persist in the peripheral blood and the organs for at least 48 h (Figure 2). In comparison, however, such induction of cytokines returns to normal levels within 24-h post lipopolysaccharide injection [39]. This suggests that “Protector” stimulatory pathway/s persist/s for longer time and/or the “Protector” active constituents may have a long half-life in the body.

It has been shown that some of the “Protector” constituents, non-water extracts, or isolated compounds exert anti-inflammatory activity in inflammatory models [30,31,32,33,34,35,36,37], reduce NF-κB or its nuclear translocation in activated T cells [30,35,36], reduce IL-6 or TNF-α following inflammatory stimulus [32,35,37]. However, in “Protector,” all the extracts were water-soluble constituents. Such water extracts should be rich in polysaccharides and polar phenolic compounds that may induce or modulate cytokines production providing distinguishable immunomodulatory activities [21,23,38,39,40]. For instance, dietary pomegranate seed oil in mice increased IgG and IgM levels [20]; calves fed pomegranate extract increased IFN-γ, IL-4 from peripheral blood mononuclear cells as well as IgG against ovalbumin vaccination [21]; polysaccharides from pomegranate extracts increased lymphocytes proliferation index [22]; grape juice diet increased circulatory γδ T cells [23], dates dietary supplements increased IgM [25]; Figs polysaccharide increased IL-12, IFN-γ, IL-6 levels [27]; and gingerols isolated from ginger increased IFN-γ from activated T cells [29]. Therefore, it may be concluded that the increase in IFN-γ and IL-12 production observed in this study could be at least due to pomegranate, figs, and ginger extracts [21,27,29]. In addition, since “Protector” oral administration increased IFN-γ and IL-12 levels in blood, spleen, and lungs following continuous daily dosing (1 vs. 3 vs. 7 days), it indicates that the oral dosing induces an additive effect on cytokines production.

In this study, we used Vaxigrip, a known commercial human viral flu vaccine, to induce the production of HAI in mice. However, it was used to test if “Protector” has an enhancing effect on the Vaxigrip-induced HAI response [43]. The present results showed that “Protector” administration increased the production of HAI following single and even more following secondary immunization. This effect shows the advantage of “Protector” when it is given with the viral flu immunization. Furthermore, Vaxigrip vaccine induced similar cytokine patterns following primary or secondary immunizations by stimulating the production of IFN-γ, IL-12, IL-6 levels in the blood, IFN-γ and IL-12 in spleen and lungs but did not modulate IL-6 in spleen or lungs. When “Protector” was administered with the vaccination, it strengthened Vaxigrip-induced cytokine responses in the primary vaccination and reversed the pro-constructive IL-22 level in the lungs. In the secondary immunization, however, “Protector” reduced IL-6 production levels and did not increase IL-12 levels in the organs tested which may suggest its regulatory pathways during successive inflammatory insults.

Viral flu infects the epithelial cells of the lungs, and within hours it induces proteins that block the innate immune system. Such blocking pathway prevents viral RNA from being recognized by PRPs, and this lowers IFN-related genes activation [18]. Recently, however, a study has demonstrated that the pre-immune state of chicken embryo fibroblast expressing IFN-γ gene inhibited the replication of viral RNA of H1N1 or H9N2 and IFN-stimulated genes were up-regulated. This effect was suppressed with siRNA for IFN-γ gene [10]. Furthermore, cytokine profile was assessed in human adults who were hospitalized due to seasonal and pandemic H1N1 influenza adults [15]. It has been shown that plasma IFN-γ level is suppressed and less frequently detected in the severe pH1N1pneumonia group whereas the patients demonstrated hyperactivation of proinflammatory cytokines mainly IL-6, and such high levels of IL-6 correlated with ICU admission [15]. Therefore, enhancing IFN-γ levels either before or at an early stage of flu infection is essential to eradicate flu virus and also reduce or control the hyperactivation of IL-6 which helps in the management of severe cases of infections.

In conclusion, “Protector” administration reinforces the protective immune parameters against viral flu infection. Therefore, after performing preclinical toxicology studies and ensuring its safety, “Protector” should be considered a potential product to be tested in clinical trials to conclude its efficacy in reducing the devastating effects of flu infection in humans and its outbreaks.
